# Krüppel-like factor 17, a novel tumor suppressor: its low expression is involved in cancer metastasis

**DOI:** 10.1007/s13277-015-4588-3

**Published:** 2015-12-12

**Authors:** Shan Zhou, Xiaowei Tang, Faqing Tang

**Affiliations:** 1 Medical Research Center and Clinical Laboratory, Zhuhai People’s Hospital and Zhuhai Hospital of Jinan University, 79 Kangning Road, Zhuhai, 519000 Guangdong China; 2 Metallurgical Science and Engineering, Central South University, 21# Lushan South Road, Changsha, 410083 China

**Keywords:** Krüppel-like factor 17, Cancer metastasis, Epithelial-mesenchymal transition, TGF-β pathway, p53 pathway

## Abstract

Krüppel-like factor (KLF) family is highly conserved zinc finger transcription factors that regulate cell proliferation, differentiation, apoptosis, and migration. KLF17 is a member of the KLF family. Recent studies have demonstrated that KLF17 low expression and inactivation are caused by microRNA, gene mutation, and loss of heterozygosity in human tumors, which participates in tumor progression. KLF17 low expression increases cancer metastatic viability; its mechanism is that low KLF17 mediates epithelial-mesenchymal transition (EMT) through regulating EMT-related genes expression; the reduced-KLF17 also increases cancer metastasis though upregulating inhibitor of DNA binding 1 (ID1). Additionally, mutant p53 proteins are capable of developing a complex with KLF17, which mediate the depletion of KLF17 inhibiting EMT gene transcription and increases cancer metastasis. KLF17 downregulation also mediates the activation of TGF-β pathway.

## Introduction

Krüppel-like factor (KLF) family is highly conserved zinc finger transcription factors, which are critical regulator of essential biological cellular processes, including proliferation, differentiation, apoptosis, and migration [1–3]. Structurally, the C-terminal region of the KLF family members is highly conserved, which is composed of triple tandem zinc fingers evenly spaced by conserved linker regions, while the N-terminal regions are highly divergent [4]. Accordingly, KLF members can recognize similar target sequences, while their N-terminus can bind to different factors leading to diverse functions [2, 4]. To date, 17 members of the KLF protein family, KLF1–17, have been described in mammals, and an increasing number of studies have demonstrated that KLF 1–17 are involved in the pathobiology of tumor progression [1, 5, 6]. KLF17, also known as zinc finger protein 393 (Zfp393), was first identified as a germ cell-specific gene in mouse [7]. van Vliet and colleagues [8] identified and renamed the hypothetical protein FLJ40160 as KLF17; KLF17 is the human homologue of murine Zfp393, and *KLF*17 gene is mapped to chromosome 1p34.1. They reported that KLF17 was a novel member of the Sp/KLF family of transcription factors and was more closely related to the KLF subfamily. Sharing similarity with *Drosophila* Krüppel gene, human Sp/KLF family is characterized by a triple-C2H2 DNA-binding domain [9, 10]. Recently, many reports have focused on KLF17 functions in tumorigenesis and found that KLF17 plays an important role in cancer development. In the present study, we summarized KLF17’s function in cancer process and its mechanism. KLF17 is downregulated and correlated with tumor progression in various human cancers. Recent studies have demonstrated that low KLF17 is involved in transforming growth factor β (TGF-β) pathway and p53 pathway in human cancer and regulates epithelial-mesenchymal transition (EMT) and participates in metastases.

## The low expression of KLF17 is involved in tumor process

### KLF17 lowly expresses in human tumors

KLFs are a family containing highly conserved zinc finger transcription factors, which contains 17 members KLF1–17 in. *KLF* family genes are mapped to chromosome 1p34.1. The short arm of human chromosome 1 is one of the most studied genomic intervals in human cancer; allelic deletions in the 1p36 and 1p32 regions correlate with poor survival [11]. KLF6 gene is mutated in a subset of human prostate cancer and involved in human prostate cancer [12]; it is also inactivated by loss of heterozygosity (LOH) [13]. Additionally, Evi-1 oncoprotein binds to the zinc finger gene and regulates KLFs’ gene expression [14]. HBx also binds to the zinc finger transcription factor and inactivates KLF gene expression in *Escherichia coli* [15]. KLF17 deficiency in tumors may also be from gene mutation and oncoprotein or virus protein inactivating. KLF17 has a transactivation activity both in embryonic chickens and humans [8, 16]. It is demonstrated that KLF17 is frequently downexpressed in the majority of human cancers, including breast cancer, lung adenocarcinoma, hepatocellular carcinoma (HCC), gastric cancer, papillary thyroid carcinoma (PTC), and non-small cell lung cancer (NSCLC) [17–22]. The expression level of KLF17 in lung adenocarcinoma cells and primary tumor tissues was lower than in immortal human bronchial epithelial cells and tumor-adjacent lung tissues, respectively [17]. The survival rate is higher in the high KLF17 expression group than in the low KLF17 expression group of patients with HCC, and the downregulated KLF17 expression is associated with the poor prognosis of HCC [18]. Peng and colleagues [20] reported that the expression level of KLF17 was significantly decreased in 98 of 158 gastric adenocarcinoma cases. Expression of KLF17 is also decreased in PTC tissues compared with the adjacent normal tissues [21].

### Low expression of KLF17 contributes to cancer cell phenotype

The forced expression of KLF17 leads to the inhibition of cell growth [23]. Silencing of KLF17 increases the transcription of CD44, plasminogen activator inhibitor 1 (PAI-1), and Cyclin-D1, while overexpression of KLF17 decreases the transcription of these genes [19]. Moreover, overexpression of KLF17 leads to cellular morphological changes and inhibits cell invasion significantly [24]. The repressed KLF17 promotes the motility and proliferation of human thyroid cancer TPC1 cells by altering the expression of zona occludens-1 (ZO-1) and Snai1, and activating the Akt pathway by upregulating inhibitor of DNA binding 1 (ID1) [21]. Low KLF17 promotes cell viability and decreases apoptosis [19]. Additionally, normal expression of KLF17 functions by directly binding to the promoter region of ID1 to inhibit its transcription, while low KLF17 expression and decreasing its inhibition to ID1 increase cell invasion and EMT shift [23]. Taken together, these findings indicate that repressed KLF17 is associated with cancer cell phenotype transition and contributes to cancer progression.

### KLF17 expression predicts survival and is associated with tumor progression

The reduced expression of KLF17 is an independent prognostic indicator of the majority of human tumors, and it is significantly associated with tumor progression. Low expression of KLF17 is also an independent predictor of lymph node metastasis in breast cancer [23]. The clinical studies showed that low KLF17 is associated with a reduced survival time in lung adenocarcinoma patients, and the distant tumor metastasis is significantly increased [17]. KLF17 expression level is an independent prognostic indicator, and it is correlated with the tumor stage and size in lung adenocarcinoma and HCC [17, 18]. It has been identified that reduced expression of KLF17 is strongly associated with tumor size, pN stage, and lymphovascular invasion in gastric adenocarcinoma [20]. Moreover, KLF17 expression is an independent prognostic factor for both overall survival and disease-free survival in gastric adenocarcinoma [20]. KLF17 expression is correlated with clinical-pathological parameters and affects the prognosis of PTC patients [21].

## KLF17 is involved in cancer metastasis

### KLF17 low expression in the metastatic cancer

The first research on KLF17 in breast cancer conducted by Gumireddy et al. [23] in 2009 reported that lower expression of KLF17 was involved in breast cancer metastasis; lower expression of KLF17 was found in breast cancer cell lines with an invasive phenotype, and lower expression of KLF17 was found in the patients with lymph node metastases compared in the patients without metastases. Repressed KLF17 is also found in metastatic HCC [18]; KLF17 is post-transcriptionally inhibited by microRNA-9 (miR-9) in HCC and implicated in miR-9-mediated HCC metastasis [25]. Low KLF17 expression is significantly associated with metastasis in lung adenocarcinoma, gastric cancer, PTC, and NSCLC [17, 20–22]. Gastric cancer studies showed that the reduced expression of KLF17 protein is correlated with its lymphovascular invasion [20]. In lung adenocarcinoma, low expression of KLF17 is also related to tumor growth and poor prognosis [17]. The downregulation of KLF17 may play a role in initiation and/or progression as well as the metastasis of esophageal squamous cancer [26]. These data indicate that KLF17 lowly expresses in metastatic tumor.

### KLF17 low expression increases cancer metastatic viability

Cancer metastasis is a complex and multistep process, which consists of a series of discrete biological processes including malignant cell spread from the primary tumor to distant foci and subsequently adaptation to distant tissue environments [27–29]. Gumireddy et al. reported that the silence of KLF17 increased cell viability in metastatic breast cancer [23]. Further studies elucidated that the low expression of KLF17 was associated with carcinoma progression, and suppression of KLF17 expression promotes tumor cell migration, invasion, and EMT shift [19, 23, 30]. KLF17 exerts its tumor suppressor function by interacting with the promoters of EMT-related genes; it was at first identified as a novel tumor suppressor from a forward genetic screen in a mouse model [23]. In recent years, a growing number of studies have demonstrated that the repressed expression of KLF17 contributes to metastasis. However, KLF17 has not always been shown to suppress metastasis, which implies that it may exist as a context dependence of suppressive pathways [31].

### KLF17 is a negative regulator of EMT

Uncontrolled cell survival, growth, angiogenesis, invasion, and metastasis are essential hallmarks of cancer [32]. Metastasis is the primary cause of cancer deaths, including a complex multistep process. In the past decades, a growing number of studies have demonstrated that EMT plays a critical role in promoting metastasis [33, 34]. KLF17 is one of the negative regulators of EMT and metastasis via regulating EMT-related genes such as E-cadherin, ID1, ZO-1, β-catenin, Snai1, vimentin, and fibronectin [5, 35]. KLF17 is the human orthologue of murine Zfp393 and can activate transcription from CACCC-box by binding to a typical G/C-rich site via its zinc fingers [8].

Epithelial cells are connected laterally via several types of cellular junctions, including adherens junctions, desmosomes, and tight junctions [36]. In addition, the basal epithelial cells are firmly anchored to the underlying basement membrane via hemidesmosomes to maintain their apical-basal polarity. Altered local microenvironment and gene promote the malignant conversion of epithelial cells to activate the EMT process. Biomarkers for EMT include the increased expression of transcription factors and proteolysis [36, 37]. When EMT shift, epithelial cells lose epithelial characteristics and acquire mesenchymal characteristics, and transdifferentiate into motile mesenchymal cells, which is essential to allow carcinoma cells to lose cell-cell junctions and depart from each other for single-cell migration and invasion [36, 38]. This switch of this program is mediated by key transcription factors, including Snail, zinc finger E-box-binding (ZEB), and basic helix-loop-helix (bHLH) transcription factors, the functions of which are finely regulated at the transcriptional, translational, and post-translational levels [33, 39–41]. The decreased level of KLF17 was correlated with reduced survival span, and the expressions of EMT-related genes were altered in HCC patient. In addition, KLF17 inhibits HCC cell invasion and migration possibly via counteracting EMT [18, 25].

Gumireddy et al. [23] reported that knockdown KLF17 led to apparent migratory phenotype, including spindle-like and fibroblastic morphology, the major characteristics of EMT in both mouse breast and human breast cancer cells. After the knockdown of the expression of KLF17, breast cancer cells displayed the major characteristics of EMT. In addition, significantly reduced expression of epithelial markers was found in KLF17 knockdown cells, while the dramatic increase of mesenchymal markers was found in KLF17 knockdown cells [23]. KLF17 can inhibit the transcription of ID1, which is the gene of encoding a key metastasis regulator in breast cancer, via directly binding to its promoter region [23]. When transfected by siKLF17, the expression of EMT-related genes, E-cadherin, ZO-1, and vimentin, changed dramatically in HepG2 cells [18]. Sun et al. [25] found that KLF17 can bind directly to the promoter regions of ZO-1, vimentin, and fibronectin and regulate ZO-1, vimentin, and fibronectin expression, so thought that ZO-1, vimentin, and fibronectin are downstream gene targets of KLF17.

## Dyregulated expression KLF17 activates signaling transduction

### miR-9 mediates KLF17 low expression

**Fig. 1:**
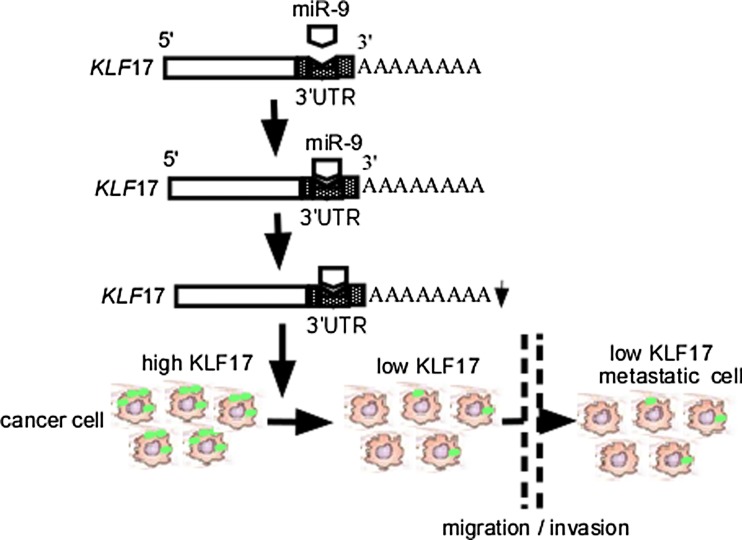
Schematic illustration of miR-9-mediated KLF17 low expression. miR-9 downregulates KLF17 expression through binding to 3’ UTR of *KLF*17 gene, increases cell migration and invasion. KLF17, Krüppel-like factor 17; 3′UTR, 3′ untranslated region; miR-9, microRNA-9

As an upstream regulator of KLF17, miR-9 inhibits KLF17 expression via directly targeting its 3′ untranslated region (3′ UTR), resulting in migration and invasion in HCC (Fig. 1). miR-9 upregulation facilitates tumor progression in diverse human cancer, including HCC [42], Hodgkin lymphoma (HL) [43], breast cancer [44], cervical cancer [45], colon cancer [46], acute myeloid leukemia (AML) [47], and gastric cancer (GC) [48]. In contrast, miR-9 undergone hypermethylation-associated silencing is correlated with metastasis in various cancers, including colorectal cancer (CRC) [49], clear cell renal cell carcinoma (ccRCC) [50], lung cancer [51], neuroblastoma [52], and nasopharyngeal carcinoma (NPC) [53]. miR-9 is regulated by prospero homeobox 1 (PROX1), a tumor suppressor. Lu and colleagues [46] further confirmed that PROX1 promotes EMT by inhibiting E-cadherin via binding to miR-9 promoter in colon cancer cells. Taken together, these studies show that miR-9 could act as an oncogene and promote the progression of HCC via KLF17.

### Low KLF17 mediates ID1 increase

**Fig. 2:**
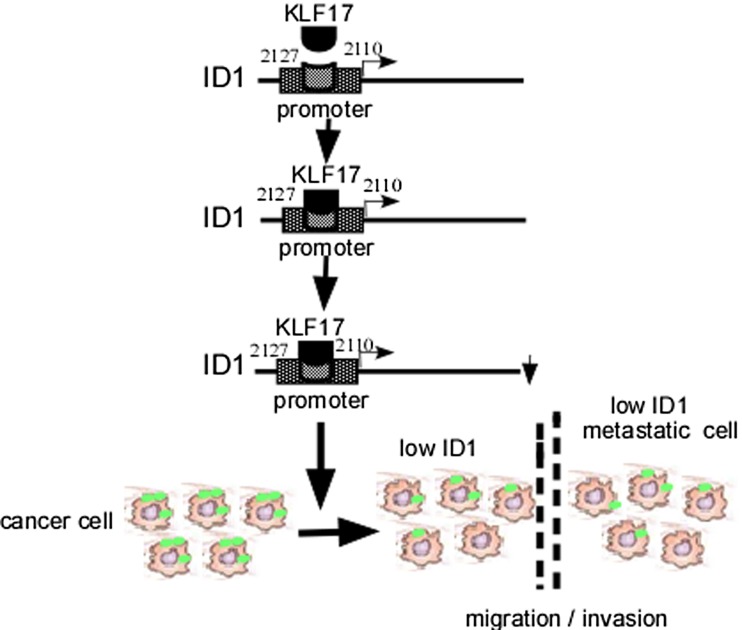
Schematic illustration of KLF17-mediated ID1 low expression. KLF17 downregulates ID1 expression through binding to *ID*1 gene promoter and increases cell migration and invasion. *ID1*, inhibitor of DNA binding 1

ID1 is one member of the vertebrate inhibitors of differentiation family and a negative regulator of bHLH transcription factors. ID1 expression exhibits a unique spatio-temporal pattern during development and malignancy [54, 55]. ID1 has been shown to play a critical role in the diverse biological process, including cell cycle, proliferation, apoptosis, senescence, and metastasis [56–58]. Gumireddy reported that KLF17 could directly bind to the mouse ID1 promoter region at −2127 to −2110 from the transcriptional initiation site and suppress the expression of ID1 [23] (Fig. 2). Elevated levels of ID1 protein have been reported in a variety of human cancers and are capable of promoting invasion and metastasis [58–60]. On the other hand, the expression of ID1 is significantly correlated with high grade and poor prognosis of human cancer [61]. ID1 plays a critical role in tumor maintenance of high-grade glioma (HGG), and deletion of ID1 and ID3 in vitro reduces invasiveness of HGG [62]. ID proteins are able to interact with bHLH proteins as homodimer or heterodimer and negatively regulate bHLH proteins [63]. Overexpression of ID1 enhances metastatic potential human thyroid tumors, which lets the thyroid tumor cells acquire the mesenchymal features [64]. Moreover, Gumireddy and colleagues found that ID1 interacted with transcription factor AP-2α (TFAP2A) to suppress S100A9 expression, leading to migratory and invasive phenotypes of cancer cells [65]. ID1 is also able to inhibit mp53-mediated endothelial cell migration and tube formation [66]. ID1 upregulates mouse double minute 2 (MDM2) expression, a key negative regulator of p53, in esophageal cancer cells [67]. Cooperating with oncogenic Ras, ID1 triggers metastatic transformation of mammary carcinoma [68]. By inducing the expression of matrix metalloproteinase (MMP) 9, ID1 promotes the invasiveness of breakpoint cluster region/Abelson (BCR/ABL) leukemia cells [69]. The overexpression of ID1 induces a significantly increased secretion of the active form of MMP2 [70] and upregulates vascular endothelial growth factor (VEGF) to promote angiogenesis in prostate cancer [71]. Additionally, ID1 can downregulate zinc finger binding protein 89 (ZBP-89), leading to mesenchymal markers’ expression and finally promoting NSCLC metastasis [72]. Taken together, these studies indicate that suppressed KLF17 in human cancers promotes metastasis through inducing ID1 (Fig. 2).

### KLF17 is a downstream mediator of the TGF-β signaling pathway

**Fig. 3:**
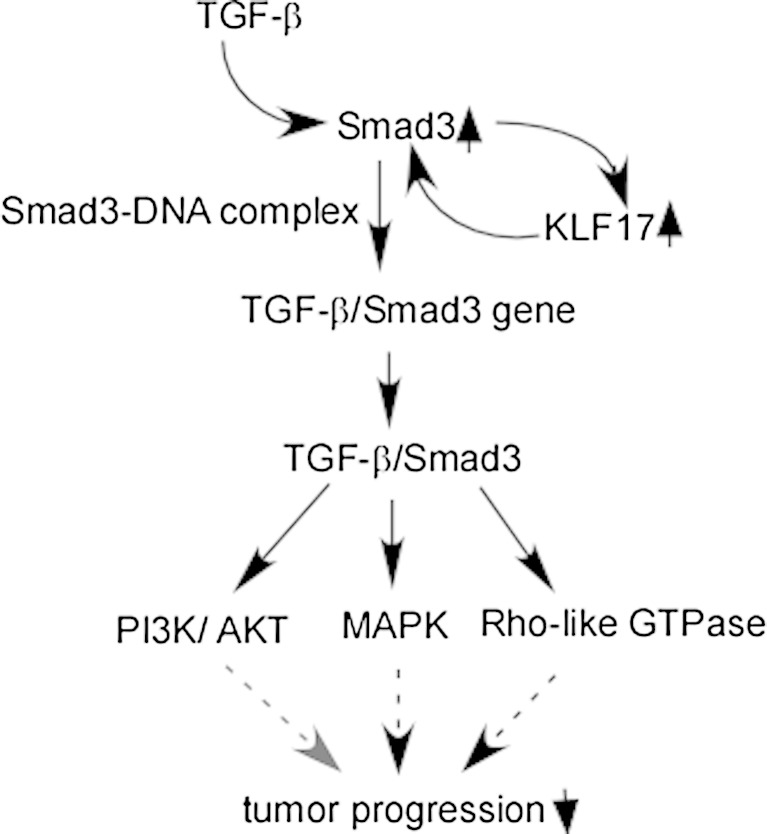
TGF-β signaling pathway mediates KLF17 expression. TGF-β enhances KLF17 expression through Smad3 as well KLF17 induces Smad3 to generate a positive feedback loop, regulates a panel of TGF-β/Smad3-dependent target genes by modulating Smad3-DNA complex formation, and finally decreases tumor progression though PI3K/AKT, MAPK, and Rho-like GTPase. *TGF-β* transforming growth factor β, *PI3K* phosphatidylinositol-3-kinase, *MAPK* mitogen-activated protein kinase

TGF-β pathway is one of the most deregulated pathways, which is intimately associated with the induction of EMT during heart development, renal fibrosis, and cancer [73–75]. TGF-β signaling can switch breast cancer cells from cohesive to single-cell motility and ultimately contribute to metastasis [76]. A recent study shows that KLF17 is a key regulator of TGF-β signaling pathway, and it has the capacity to suppress tumor progression through potentiating TGF-β/Smad3-dependent signaling pathway [30]. TGF-β enhances KLF17 expression in multiple cancer cells via Smad3; KLF17 induces Smad3 to generate a positive feedback loop, which regulates a panel of TGF-β/Smad3-dependent target genes by modulating Smad3-DNA complex formation [30] (Fig. 3). So far, three types of Smads have been identified, such as receptor-regulated Smads (R-Smads), common-partner Smads (Co-Smads), and inhibitory Smads (I-Smads) [77]. In the canonical pathway, R-Smads are directly phosphorylated by the activated type I TGF-β receptors (TβRI). Receptor-mediated phosphorylation of R-Smads together with Smad4 induces their accumulation in the nucleus, where they interact with other transcription factors to regulate transcriptional responses [73, 78]. As a tumor suppressor pathway, TGF-β signaling is well illustrated by modulation of receptors and Smads in cancers, which is further supported by studies of cancer development in mouse models [79]. In the non-canonical pathway, TGF-β signal is transducted via three pathways including mitogen-activated protein kinase (MAPK) pathways, Rho-like GTPase signaling pathways, and phosphatidylinositol-3-kinase (PI3K)/Akt pathways [80] (Fig. 3).

### KLF17 functions via p53-dependent pathway

**Fig. 4:**
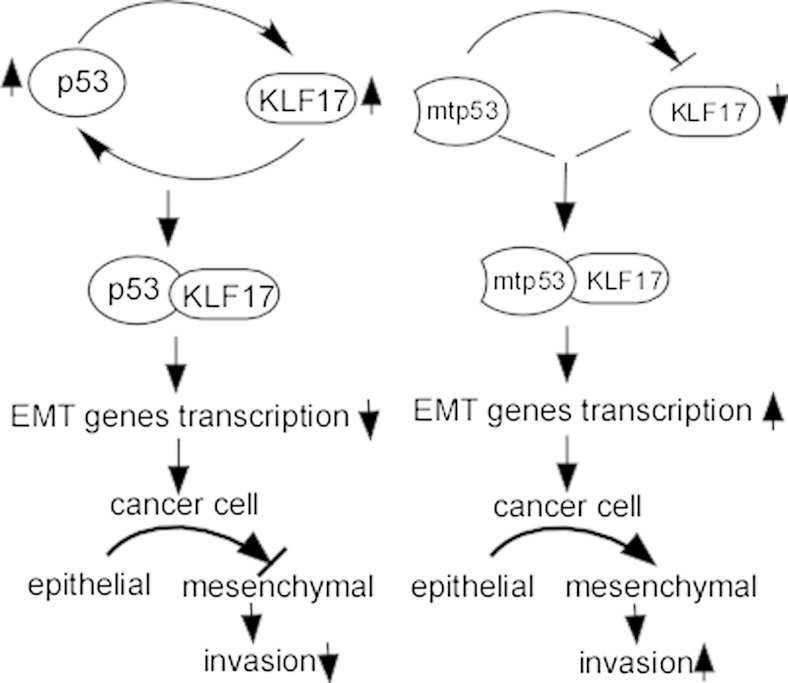
Mutant p53-mediated cancer invasion through inhibiting KLF17. p53 enhances KLF17 transcription and KLF17 and enhances p53 transcription to generate a positive feedback loop. Mutant p53 potentiates cancer progression through inhibition of KLF17 expression. Mutant p53 protein develops a complex with KLF17, leading to inhibit KLF17-mediated EMT gene transcription. *mtp53* mutant p53, *EMT* epithelial-mesenchymal transition

Wild-type p53, a major tumor suppressor, participates in diverse cellular stress stimuli, including ribosomal stress, nutrient depletion, viral infection, oncogenes activation and hypoxia, and heat shock [81, 82]. As a critical regulator of metastasis, p53 directly regulates the transcription of metastatic genes including EMT and stemness genes, interacts with ECM and anoikis, and inhibits cancer metastasis [83] (Fig. 4). However, mutant p53 loses tumor suppressor activity and gains functions, and contributes to malignant progression [84–86]. Recent studies have shown that KLF17 is involved in p53 pathway. Ali and colleagues [22] showed that KLF17 exerted an anti-EMT effect via the p53-dependent pathway in NSCLC. KLF17 mRNA levels were induced in a dose-dependent manner in siRNA targeting p53 A549 cells with Nutlin-3 treatment, but there was not KLF17 transcription in p53 depleted cells [22]. p53 enhances KLF17 transcription, and KLF17 enhances p53 transcription to generate a positive feedback loop (Fig. 4). Furthermore, p53 interacts with KLF17 promoter via p53 consensus responsive element (p53RE) and recruits p300 in response to chemotherapy [22]. In contrast, mutant p53 potentiates cancer progression through KLF17 inhibition via recruiting to the upstream of KLF17 promoter in metastatic breast cancer. In addition, endogenous mutant p53 proteins are capable of developing a complex with KLF17, which inhibits KLF17-mediating EMT gene transcription (Fig. 4). The metastasis suppressor ability of KLF17 to EMT target genes is enhanced when mutant p53 is depleted [19]. Taken together, these studies suggest that suppressed KLF17 leads to weaken the tumor suppressor strength of p53.

#### KLF17 may be a downstream signaling molecule of DJ-1

**Fig. 5:**
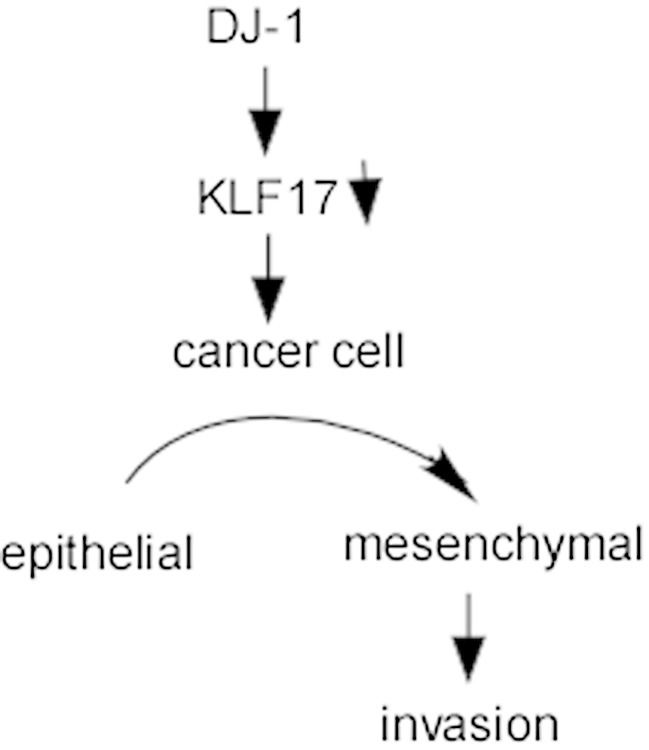
Schematic illustration of DJ-1-mediated low KLF17 expression. DJ-1 decreases KLF17 expression, promotes cell transition from epithelial to mesenchymal, and increases cell invasion. *DJ-1* parkinson protein 7

DJ-1 [anti-oxidant protein encoded by *PARK7* gene (Parkinson protein 7 gene)] is a conserved protein ubiquitously expressed in human tissues, which is a human oncogene identified in 1997 [87]. DJ-1 is overexpressed in a variety of human cancers and correlated with poor prognosis [88]. The increased levels of DJ-1 are detected in the nipple fluid of breast cancer patients [89]. It has been shown that DJ-1 is also implicated in multiple cellular processes, including cell proliferation, invasion, and metastasis [90, 91]. Recently, it is reported that DJ-1 is highly expressed in invasive breast cancer cell and is able to repress the expression of KLF17 to promote breast cancer cell invasion by downregulating E-cadherin and increasing Snail expression (Fig. 5); moreover, DJ-1 could directly regulate KLF17 by binding to the ID1 promoter [24]. Taken together, DJ-1 could promote the invasion of breast cancer cells via regulating the KLF17/ID1 pathway.

## Conclusions

Since KLF17 was at first identified as a tumor suppressor, an increasing number of studies have reported that KLF17 is frequently downregulated, which is correlated with tumor progression in various human cancers. KLF17 low expression promotes metastasis; its mechanism is to directly increase cell invasion and initiate EMT shift though regulating EMT gene expression. Additionally, the reduced-KLF17 in human cancer is involved in TGF-β pathway and p53 pathway. However, tumor metastasis is a result of many genes’ concerted action, the precise mechanism of KLF17-involved metastasis is still incomplete, and much more challenges for researchers need to be overcome.
